# Thrombotic Antiphospholipid Syndrome Shows Strong Haplotypic Association with *SH2B3-ATXN2* Locus

**DOI:** 10.1371/journal.pone.0067897

**Published:** 2013-07-03

**Authors:** Eguzkine Ochoa, Mikel Iriondo, Ana Bielsa, Guillermo Ruiz-Irastorza, Andone Estonba, Ana M. Zubiaga

**Affiliations:** 1 Department of Genetics, Physical Anthropology and Animal Physiology. School of Science and Technology, University of the Basque Country (UPV/EHU), Leioa, Spain; 2 Autoimmune Disease Research Unit, Service of Internal Medicine, Hospital de Cruces, University of the Basque Country (UPV/EHU), Barakaldo, Spain; National Institute of Environmental Health Sciences, United States of America

## Abstract

**Background:**

Thrombotic antiphospholipid syndrome is defined as a complex form of thrombophilia that is developed by a fraction of antiphospholipid antibody (aPLA) carriers. Little is known about the genetic risk factors involved in thrombosis development among aPLA carriers.

**Methods:**

To identify new loci conferring susceptibility to thrombotic antiphospholipid syndrome, a two-stage genotyping strategy was performed. In stage one, 19,000 CNV loci were genotyped in 14 thrombotic aPLA+ patients and 14 healthy controls by array-CGH. In stage two, significant CNV loci were fine-mapped in a larger cohort (85 thrombotic aPLA+, 100 non-thrombotic aPLA+ and 569 healthy controls).

**Results:**

Array-CGH and fine-mapping analysis led to the identification of 12q24.12 locus as a new susceptibility locus for thrombotic APS. Within this region, a *TAC* risk haplotype comprising one SNP in *SH2B3* gene (rs3184504) and two SNPs in *ATXN2* gene (rs10774625 and rs653178) exhibited the strongest association with thrombotic antiphospholipid syndrome (p-value = 5,9 × 10^−4^ OR 95% CI 1.84 (1.32–2.55)).

**Conclusion:**

The presence of a *TAC* risk haplotype in *ATXN2-SH2B3* locus may contribute to increased thrombotic risk in aPLA carriers.

## Introduction

Antiphospholipid syndrome (APS) is a complex autoimmune disease characterized by the presence of antiphospholipid antibodies (aPLA) along with the development of thrombosis and/or pregnancy morbidity [1,2,3]. It is thought that aPLAs are able to interact with hemostatic and inflammatory mediators, giving rise to the pro-coagulant/pro-thrombotic manifestations that characterize APS [4,5]. However, only a fraction of individuals with elevated aPLA titers develop thrombosis (thrombotic APS), suggesting that additional risk factors may be involved in thrombosis development in these individuals.

Gene expression profiling at the transcriptome and the proteome level has confirmed the link in APS between immune responses and coagulation pathways [6,7,8,9], but hasn’t clarified which genes could be responsible for the development of thrombotic APS. At the genomic level, genetic variants that confer susceptibility to aPLA production and APS development have been widely investigated in recent years. Genetic association studies based on candidate genes have shown significant association of polymorphisms involved in blood coagulation (*F5, F13A1*) and proinflammatory state (*TLR4*) with thrombotic APS [10,11,12,13]. Despite the progress achieved by these studies, the collected body of data is still insufficient to distinguish individuals who will develop thrombotic events from those who will not.

In recent years, copy-number variants (CNV) have emerged as genomic variants that may contribute to the genetic basis of human disease susceptibility [14]. It has been estimated that 12% of the human genome is composed of such sequences, and their presence can change gene dosage, cause protein diversity and/or allow the evolution of new functions [15]. Some copy number variants have been associated with immune-related traits. For example, the presence of a CNV in the *FCGR3B* gene has been linked to the development of glomerulonephritis in patients with systemic lupus erythematosus [16,17]. Recently, a study directed by the Wellcome Trust Case Control Consortium (WTCCC) has discovered several CNV loci that are associated with common diseases, such as coronary artery disease, type 2 diabetes, hypertension or rheumatoid arthritis [18]. Importantly, numerous CNVs identified in this study co-localized with SNPs that had been previously reported in genome-wide association (GWA) studies, suggesting that disease susceptibility regions might harbor genomic variants at an elevated frequency.

In this report, we have searched for new susceptibility loci for thrombotic APS. By performing a combination of array-CGH and SNP-based association analyses we have identified the 12q24.12 locus as a new susceptibility region for thrombotic APS. The identification of this susceptibility locus could contribute to our understanding of the molecular basis of thrombotic APS and could help in the clinical management of patients affected by this disorder.

## Materials and Methods

### Study Cohort

All subjects included in the study were Spanish Caucasian individuals. Samples from cases were collected at the Autoimmune Disease Research Unit of Hospital de Cruces (Barakaldo, Spain) during years 2008–2010. Samples from healthy controls were collected at the Basque Biobank for Research-OEHUN (Spain). The protocols for human subjects’ recruitment and study were approved by the ethical board (institutional review board) of Hospital Universitario Cruces (Barakaldo, Spain). Samples and data from patients were provided by the Basque Biobank for Research-OEHUN (www.biobancovasco.org) and were processed following standard procedures with appropriate ethical approval. All subjects were informed about the study design and goals, and signed the informed consent. Genomic DNA was extracted from whole blood with Flexigen kit (Qiagen Inc, California, USA) at the Basque Biobank for Research-OEHUN. DNA concentration was measured using a NanoDrop Spectrophotometer (NanoDrop Technologies, Inc, Wilmington, DE).

For CNV association analyses (stage 1), we selected Spanish Caucasian patients with high aPLA titers and severe thrombotic manifestations (aPLA+/th+, n = 14) and sex and ethnicity-matched Spanish Caucasian healthy controls (controls; n = 14) without family history of autoimmune diseases (Table 1). We considered a severe thrombotic phenotype when an individual had suffered more than one thrombotic manifestation.

**Table 1:** Characteristics of individuals included in the study.

Analysis	Group	N	Gender (% Females)	Age at inclusion (Years)
Array-CGH	aPLA+/th+	14	50.00%	44.64±11.6
	Controls	14	50.00%	44.50±9.80
Fine-mapping	aPLA+/th–	100	85.00%	50.3±15.1
	aPLA+/th+	85	63.30%	51.3±14.2
	Controls	569	53.20%	43.2±10.4

For SNP association analyses (stage 2), samples were collected from Spanish Caucasian individuals with high anti-phospholipid antibody titers (aPLA+; n = 185) and healthy Spanish Caucasian individuals without family history of autoimmune diseases (controls; n = 569) (Table 1). To be considered in the aPLA+ case group, individuals had to exhibit elevated anti-phospholipid antibody levels on at least two occasions twelve weeks apart [19]. In the aPLA+ group we distinguished two subsets: non-thrombotic (aPLA+/th-, n = 100) and thrombotic (aPLA+/th+, n = 85). The non-thrombotic group included APS patients exhibiting obstetric complications, patients with systemic lupus erythematosus and high aPLA titers, and asymptomatic individuals with high aPLA levels [2]. The thrombotic group included patients with primary or secondary APS along with one or more thrombotic manifestations [2]. Therefore, we carried out 3 types of comparisons in stage two: aPLA+/th+ individuals *vs.* healthy controls; aPLA+/th- individuals *vs.* healthy controls; and aPLA+/th+ *vs.* aPLA+/th- individuals.

### Study design

**Figure 1:**
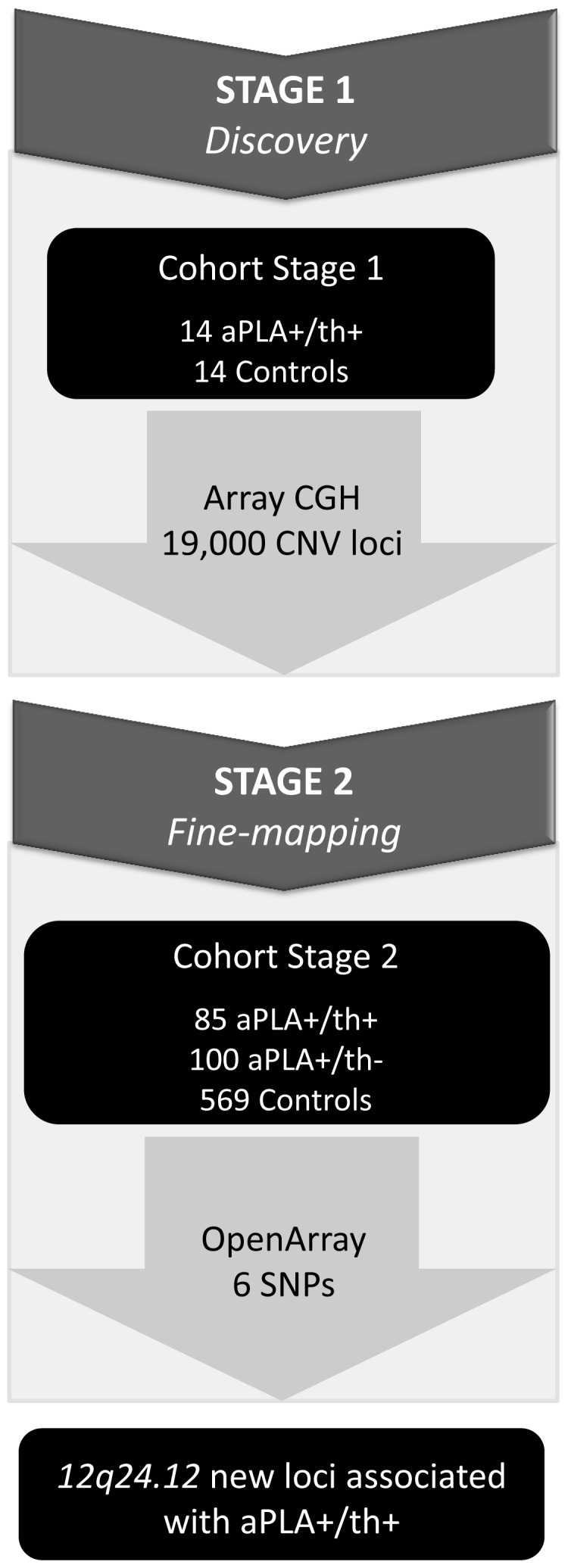
Overview of study design.

A two-stage genotyping strategy was performed to identify new susceptibility regions associated with thrombotic aPLA carriers (Figure 1). In stage one, 19,000 CNV loci were genotyped in 14 aPLA+/th+ individuals and 14 healthy controls. In stage two, CNV loci associated with thrombotic APS were fine-mapped. Several criteria were considered for CNV selection: (i) to be located in regions with suggestive association with autoimmunity and cardiovascular diseases, as demonstrated by array-CGH (FDR<0.20) and published genome-wide association studies (GWAS) (*p*-value≤5 × 10^−8^) (ii) to lie outside CpG islands; (iii) to exhibit a sequence length <5kb; and (iv) to know their minor allele frequencies (MAF).

### Genotyping

In the discovery stage, screening of genome-wide copy number variants were carried out by array-based comparative genomic hybridization (CGH). Human Copy Number Variation Microarray Kit 2×105K produced by Agilent (Agilent Technologies, Santa Clara, CA) and Welcome Trust Case Control Consortium (WTCCC) was the selected platform. This high-resolution 60-mer oligonucleotide-based microarray contains more than 99,000 60-mer probes spanning coding and non-coding genomic sequences with median spacing of 244 bp respectively. This platform contains 19,000 CNVs that were identified by the WTCCC, the Toronto Database (DGV, Database of Genomic Variants) and the Sanger Consortium for the study of structural variants associated with human disease [20,21,22,23]. Array-CGH hybridization and data extraction were performed in NIMgenetics (www.nimgenetics.com, Parque Científico, Tres Cantos, Madrid). Array labeling and hybridizations were performed according to the manufacturer’s protocol for array-CGH experiments (Agilent Technologies). Arrays were examined using the DNA Microarray Scanner C (Agilent Instruments) and images for the array-CGH were extracted with Agilent Feature Extraction software (v10.5), which identified the highest quality pixels in each feature for intensity quantitation.

CNV loci were fine-mapped in stage two. For this purpose, we focused on a genetic region around each CNV, and examined the genes mapping this larger region. SNP selection was carried out by Tagger option implemented in Haploview software, v.4.3 [24]. Tagger selects a minimal set of markers, such that all alleles to be captured are correlated at an r^2^. TaqMan® OpenArray™ Genotyping System (Applied Biosystems) was used for genotyping. This system uses two allele-specific MGB probes and two PCR primers to provide highly robust and accurate genotyping calls. Data were collected by OpenArray NT Imager Software and analyzed by Taqman Genotyper Software.

### Statistical Analysis

To identify the genomic regions of interest discovered by array-CGH we used ADM-2 statistical algorithm [25] based on the combined log2 ratios. Briefly, this statistical procedure identified the regions in the genome for which the weighted average of the measured probe signals is different from the expected value of 0 by more than a given threshold. Statistical threshold of the ADM-2 algorithm is the minimum ±log2 ratio, and for the minimum number of probes in a CNV interval, we used a threshold of 6. For each region included in the array, a hit was considered positive when it was detected in at least five consecutive positive probes. Significant differences in CNV distribution between thrombotic aPLA+ carriers and healthy controls were determined by two different statistics: Fisher’s F test for the study of the qualitative values (duplication and deletion) and Student’s t test for the study of quantitative values (value of the mean of the log ratio of the region). Both statistics were corrected using False Discovery Rate (FDR) analysis.

SNP genotype data were filtered using quality parameters checked by Haploview software, v.4.3 [24]. SNPs with a call rate lower than 95%, or those with significant deviations from Hardy-Weinberg equilibrium in controls (HWE; *p*<0.001) were excluded. Individuals with a call rate lower than 90% were also excluded from the study. Allele frequencies were compared among cases and controls using chi-square analysis with 1 degree of freedom to find significant associations using PLINK software, v.1.07 [26]. p-values below 0.05 after correction by Benjamini and Hochberg FDR method were considered as statistically significant (referred to as P_FDR_). Linkage disequilibrium (LD) patterns among SNPs were calculated using Haploview v.4.3 defining the blocks by confidence intervals [27]. Association analyses at haplotype level were performed by PLINK v.1.07. Haplotypes with *p*-values <0.05 were considered statistically significant.

## Results

Genomic comparisons by array-CGH analysis detected ninety-six CNVs (FDR<0.20, supplementary table 1) with suggestive differences in distribution between thrombotic APS (aPLA+/th+; n = 14) and healthy controls (controls; n = 14). Among the identified regions, 12q24.12, 13q34 and 19p13.2, had previously shown significant association with autoimmunity and/or cardiovascular disease in genome-wide association studies (Table 2) [28,29,30,31,32,33,34,35,36,37,38,39,40]. We focused on the 12q24.12 locus and excluded 13q34 and 19p13.2 loci for further analyses after applying our selection criteria (see Materials & Methods section. Study design) (Table 2). The CNV detected at the 12q24.12 locus has a sequence length of 1,930 bp, it is located in intron 15 of the *ATXN2* gene, and shows suggestive gains in thrombotic aPLA+ individuals in comparison with healthy controls. This CNV is included in the larger CNV V_66331, previously described by Conrad and collaborators [41].

**Table 2:** Candidate susceptibility regions with positive results in array-CGH and published GWA studies.

		*Array-CGH*						*DGV* a	
Region	Gene Names	CNV (pb)	CNV type		*p*-value	FDR	CpG	CNV ID	MAF
12q24.12	ATXN2	1,930	Gain	5.4×10^−144^		0.1957	no	V_66331	0.0387
13q34	F7	3,345	Loss	2.6×10^−22^		0.0702	yes	V_66565	0.0139
19p13.2	FCER2, CLEC4G, CD209,CLEC4M, EVI5L	175,300	Gain	3.3×10^−17^		0.0702	yes	V_8863	n.a

a DGV, Database genomic variants.

**Figure 2:**
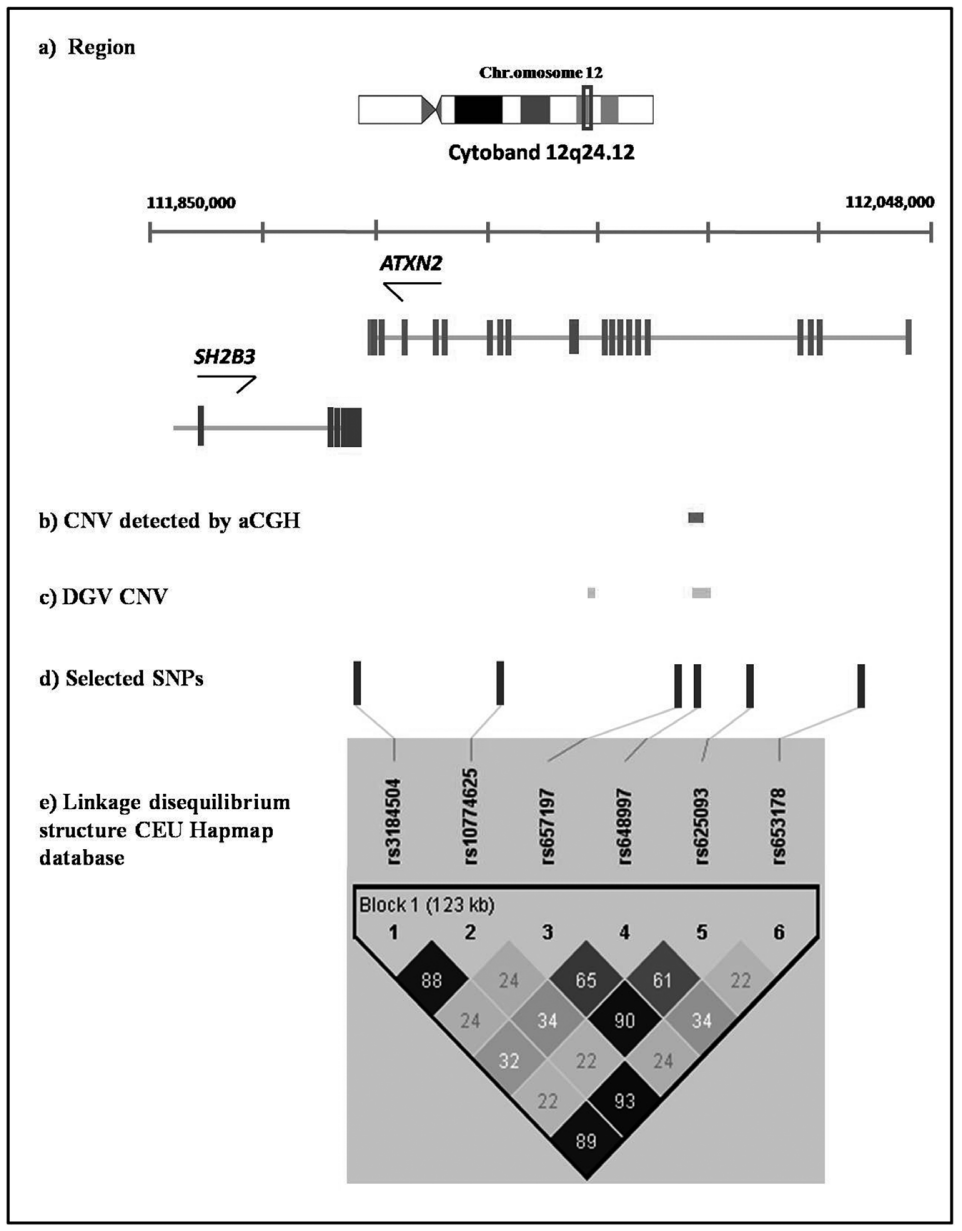
Candidate susceptibility region 12q24.12 identified by the combination of results obtained with array-CGH and published genome-wide association studies (GWAS) in related diseases. a) description of the region, b) location of the MCR identified in our array-CGH analysis, c) previously described CNVs in the region, d) SNPs selected for our genotyping analysis, e) linkage disequilibrium (LD) structure across the 12q24.12 locus. The LD structure has been obtained with Haploview software v.4.3, based on r^2^ coefficient calculated with the CEU HapMap database.

Fine-mapping of this CNV locus was subsequently performed in a larger cohort (185 aPLA+/569 controls) by SNP-based genetic association analysis. To this end, we selected six tag SNPs in 12q24.12 locus to capture as much variation in the region as possible (Table 3). Of these, one SNP lied inside the CNV (Figure 2). Five of the six SNPs fulfilled the quality control criteria (i.e. minimal call rate and HWE), and were included in the association analysis. SNP rs648997, located within the CNV (Figure 2), was initially excluded because it showed perturbation of allele frequencies and lack of HWE in healthy controls (p-value = 0.0018) and in aPLA+/th- individuals (p-value = 0.0005). However, these alterations could arise from the existence of a CNV. In fact, it is known that CNVs can perturb SNP allele frequencies at the CNV locus, which appears to violate HWE [20,22,23]. Therefore, we included SNP rs648997 in the association analysis.

**Table 3:** SNP selection in 12q24.12 candidate susceptibility region.

Chr	Gene	SNP#ID	Position	SNP	MAF Controls	SNP Type	Disease Associations
12	SH2B3	rs3184504	111884608	C/T	0.44	TagSNP Missense W262R	Celiac disease [40,53]Hypertension & Blood pressure [34]Type 1 Diabetes [54]Myocardial infarction [30]Multiple sclerosis [49]Rheumatoid arthritis [37,40]Systemic lupus erythematosus [29]
12	ATXN2	rs10774625	111910219	A/G	0.422	TagSNP	Retinal vascular caliber [31]
12	ATXN2	rs657197	111965658	G/T	0.251	TagSNP	
12	ATXN2	rs648997	111976776	A/G	0.337	TagSNP/CNV^a^	
12	ATXN2	rs625093	111988432	C/T	0.243	TagSNP	
12	ATXN2	rs653178	112007756	T/C	0.422	TagSNP	Celiac Disease [53]Hypertension & Blood pressure [34]

a CNV, copy number variant detected by array-CGH.

Two types of comparisons (aPLA+/th+ *vs*. healthy controls, and aPLA+/th+ *vs*. aPLA+/th-) showed significant association of SNPs located in *SH2B3* and *ATXN2* genes with thrombotic APS (Table 4). We found significant differences in allelic frequencies for SNP rs3184504 in *SH2B3* and SNPs rs10774625 and rs653178 in *ATXN2*. Individuals with a T allele at the rs3184504 SNP in *SH2B3* or a G allele at the rs653178 SNP in *ATXN2* had higher risk of developing thrombotic APS. In contrast, individuals with a G allele at the rs10774625 SNP in *ATXN2* exhibited higher protection from thrombotic APS. No significant associations were observed when we compared aPLA+/th- individuals with healthy controls.

**Table 4:** Significant allelic associations detected and odds ratios of SNPs.

Compared groups	SNP# ID	SNP	MAF Cases	MAF Controls*	p-value	P_FDR_	OR (95% CI)
aPLA+/th+ *vs.* controls	rs3184504	C/T	0.5655	0.4401	0.0024	0.0048	1.66 (1.19–2.30)
aPLA+/th+ *vs.* controls	rs10774625	A/G	0.5549	0.422	0.0014	0.0048	0.58 (0.42–0.81)
aPLA+/th+ *vs.* controls	rs653178	T/C	0.5602	0.4217	0.0008	0.0048	1.75 (1.26–2.43)
aPLA+/th+ *vs.* aPLA+/th-	rs3184504	C/T	0.5655	0.4271	0.0092	0.0215	1.73 (1.14–2.63)
aPLA+/th+ *vs.* aPLA+/th-	rs10774625	A/G	0.5549	0.4141	0.0081	0.0215	0.57 (0.38–0.87)
aPLA+/th+ *vs.* aPLA+/th-	rs653178	T/C	0.5602	0.415	0.006	0.0215	1.78 (1.18–2.70)

* In the aPLA+/th+ *vs.* aPLA+/th- analysis the aPLA+/th- are considered as controls for the purpose of MAF.

We next performed a haplotypic analysis to search for linkage disequilibrium (LD) patterns in the region. We found strong linkage disequilibrium with 3 out of the 6 SNPs analyzed (r^2^>0.9). The most frequent haplotype in aPLA+/th+ individuals was *TAC*, which was built from SNP rs3184504 in *SH2B3* and SNPs rs10774625 and rs653178 in *ATXN2*. The LD structure of SH2B3-ATXN2 locus in Caucasian population (*HapMap*, *CEU*) is shown in Figure 2. Haplotype frequencies between aPLA+/th+ individuals and healthy controls were significantly different (p-value = 5.89 × 10^−4^) for this locus. We also observed significant association with the *TAC* block after comparing thrombotic and non-thrombotic aPLA+ individuals (p-value = 5.94 × 10^−3^) (Table 5). In contrast, the comparison performed between aPLA+/th- individuals and healthy controls detected no significant association (p-value = 0.8329). LD pattern analysis between the associated haplotype and the CNV revealed consistent pairwise linkage disequilibrium (D′ = 1), indicating an absence of recombination for all markers in this region.

**Table 5:** Haplotype associations detected between rs3184504-rs10774625-rs653178.

Compared groups	Haplotype	Freq Cases	Freq Controls	p-value	OR (95% CI)
aPLA+/th- *vs.* controls	TAC	0.4102	0.4183	0.8329	0.96 (0.71–1.31)
	CGT	0.5787	0.5645	0.7127	1.01 (0.75–1.38)
aPLA+/th+ *vs.* controls	TAC	0.5602	0.4183	5,9 × 10^−4^	1.84 (1.32–2.55)
	CGT	0.4398	0.5645	0.002616	0.56 (0.40–0.77)
aPLA+/th+ *vs.* aPLA+/th-	TAC	0.5602	0.4102	0.00594	1.91 (1.26–2.90)
	CGT	0.4398	0.5787	0.005937	0.55 (0.36–0.83)

## Discussion

Thrombotic antiphospholipid syndrome is a complex form of antibody-induced thrombophilia. Antiphospholipid antibodies are considered a risk factor for thrombophilia, although the only presence of aPLAs is not sufficient to induce a thrombotic event. The present study was designed to find new susceptibility regions contributing to the development of thrombosis in aPLA carriers. The combination of data gathered from our array-CGH analysis, together with GWAS data that have been published previously on autoimmunity and cardiovascular diseases led to the identification of 12q24.12 locus as a new susceptibility region in thrombotic APS patients.

Our results show an elevated frequency of genomic variants (both CNV and SNPs) within the 12q24.12 locus in thrombotic APS. This region, which encompasses *ATXN2* and *SH2B3* genes, has been previously associated with several complex diseases, such as retinal vascular caliber and chronic kidney disease, myocardial infarction or type 1 diabetes [30,31,35,42]. SH2B3 (also known as LNK) is the member of a family of adaptor proteins known to negatively regulate intracellular signals delivered through the T-cell and cytokine receptors [43,44,45]. We found significant allelic and haplotypic association in this region with thrombotic aPLA carriers in comparison with healthy controls and non-thrombotic aPLA carriers. Interestingly, haplotypic association with thrombotic aPLA carriers was more significant than allelic association of each individual SNP or the presence of the CNV V_66331. The *TAC* risk haplotype, firstly described in this work, is composed of SNP rs3184504 in *SH2B3* gene and SNPs rs10774625, rs653178 in *ATXN2* gene. Even though this haplotype is common in the general population, it is significantly more frequent in thrombotic aPLA+ individuals. Taken together, our results suggest that the main genetic risk factor for thrombotic aPLA carriers at the 12q24.12 locus is the *TAC* risk haplotype, whereas the CNV itself could be considered as a tag of the associated haplotype.

SNPs rs10774625 and rs653178 of the TAC risk haplotype, mapping the *ATXN2* gene, have no known functional effect. By contrast, the SNP rs3184504, mapping exon 3 of the *SH2B3* gene, is a missense variant (p.R262W; c.784T>C). The R262W amino acid change is located in the pleckstrin homology (PH) domain of LNK, involved in plasma membrane targeting [46]. The high conservation in mammals of the R262 residue in LNK suggests that it may have functional relevance [47]. The *T* risk allele at SNP rs3184504 has been associated in a previous study on type 1 diabetes with the activation and expansion of self-reactive lymphocytes in susceptible individuals [48]. This evidence was further supported by results obtained in celiac disease [40], systemic lupus erythematosus [29], rheumatoid arthritis [37,40] and multiple sclerosis [49], underscoring the pivotal role of *SH2B3* in loss of immune tolerance and development of autoimmunity. Intriguingly, T allele carriers of rs3184504 marker also show stronger activation of the innate immune response pathway [50]. Thus, the presence of *T* risk allele of rs3184504 in thrombotic APS patients could be a contributing factor for the activation of self-reactive lymphocytes and of the innate immune responses, both of which have been described in this autoimmune disorder [7,8].

Interestingly, both *SH2B3* and *ATXN2* genes have also been associated with cardiovascular alterations, such as myocardial infarction, hypertension, blood pressure and retinal vascular caliber [30,31,32]. The functional connection between this haplotype and thrombophilia risk is still unknown, but it might also rely on the *T* allele of rs3184504 marker in *SH2B3.* The protein adaptor coded by *SH2B3* functions as a negative regulator of *TNF* signaling in endothelial cells [51] and may contribute to the progression of plaque formation in coronary arteries [52]. These observations suggest that APS individuals carrying the *T* allele in *SH2B3* could exhibit increased thrombotic risk owing to functional defects in the protein encoded by this gene. Given that *SH2B3* functions as inductor of a pro-inflammatory state in blood vessels, it would be interesting to determine whether R262W substitution in *SH2B3* contributes to the pathogenesis of autoimmunity and thrombotic phenotype in APS. Despite the limitation of our discovery cohort size and the inherent difficulties and uncertainties that the CNV genotyping and analysis pose, our work has uncovered a novel genetic susceptibility region (*ATXN2*-*SH2B3*) associated with aPLA+/th+ patients. Further studies will be required in other populations in order to confirm our findings.

## Supporting Information

Table S1 **List of CNVs exhibiting suggestive differences in distribution (FDR <0.20) between thrombotic antiphospholipid syndrome and healthy controls.**
(XLSX)
